# Rare stroke mechanisms in 4154 consecutive patients: causes, predictors, treatment, and outcomes

**DOI:** 10.1007/s10072-022-06344-w

**Published:** 2022-08-22

**Authors:** Alex Vicino, Gaia Sirimarco, Ashraf Eskandari, Dimitris Lambrou, Philippe Maeder, Vincent Dunet, Patrik Michel

**Affiliations:** 1 Stroke Center, Neurology Service, Department of Clinical Neurosciences, Lausanne University Hospital (CHUV) and University of Lausanne, Rue du Bugnon 46, CH-1011 Lausanne, Switzerland; 2 Diagnostic Neuroradiological Unit, Service of Diagnostic and Interventional Radiology, Department of Medical Radiology, Lausanne University Hospital and University of Lausanne, Lausanne, Switzerland

**Keywords:** Ischemic stroke, Rare stroke, Stroke mechanism

## Abstract

**Background:**

Rare mechanisms of stroke (RMS) in acute ischemic stroke (AIS) have rarely been studied applying a systematic approach. Our aim was to define the frequency, etiologies, predictors, and outcomes of RMS in a consecutive series of AIS.

**Methods:**

Data from consecutive patients from 2003 to 2016 were derived from the Acute STroke Registry and Analysis of Lausanne (ASTRAL). Frequency of subcategories of RMS was calculated. In a case–control design, RMS were compared to strokes of all other mechanisms. Outcome was assessed with 3-month Rankin-shift and 12-month mortality and recurrence rates.

**Results:**

Out of 4154 AISs, 222 (5.3%) were found to have a RMS (42.0% female, median age 66 years). The most frequent RMS etiologies were medical interventions (25.6%), active oncological disease (22.5%), and vasculitis (11.7%). In multivariate analysis, RMS patients were younger, had more preceding and bilateral strokes, and a higher admission temperature. They were associated with less traditional risk factors and more systemic disease (such as AIDS, coagulopathy, and cancer). RMS also had more early ischemic changes on plain CT, less revascularization treatments, and more symptomatic hemorrhagic transformations. They presented significantly higher 3-month disability (Rankin-shift-OR_adj_ 1.74), 12-month recurrence (OR_adj_ 1.99), and mortality rates (OR_adj_ 2.41).

**Conclusions:**

RMS occurred in 5.3% of a large population of consecutive AISs and are most frequently related to medical interventions, cancer, and vasculitis. RMS patients have less traditional risk factors but more systemic comorbidities, hemorrhagic transformations, recurrences, and a worse long-term outcome. Identification of RMS has direct implications for early treatment and long-term outcome.

**Supplementary information:**

The online version contains supplementary material available at 10.1007/s10072-022-06344-w.

## Introduction

Ischemic strokes originate usually from mechanisms like cardiac sources, atherosclerosis, small vessel disease, or dissection in young patients. In some patients, other causes are identified, which were labeled “stroke of other determined origin” in the original TOAST classification [1]. Multiple processes may account for such rare mechanisms of stroke (RMS), such as vasculitis (primary systemic [2–4], from an underlying systemic disease [5–7], or isolated CNS vasculitis), hypercoagulability states [8, 9], cancer-related [10], drug-related, migrainous infarction, vasospasm, intervention-related, or genetic. Only a few publications have focused on the clinical presentation, causes, and long-term outcome in large series of RMS.

In the current literature, strokes of other determined etiology were infrequent, ranging from 2.5 to 6.2% [11] [12] [13] [14]. In a previous publication focusing on RMS [14], such strokes seemed to occur more often in younger patients with less cardiovascular risk factors. In addition, the most represented etiology group was hematological disease, followed by infections, migrainous stroke, vasculitis, and a “miscellanea” group. Their short-term clinical outcome was better than in patients with other acute cerebrovascular events.

The aim of our study was to define the frequency, etiologies, predictors, and outcomes of RMS in a large consecutive single-center series of AIS. Such knowledge could help to suspect a RMS early in the disease course, to prompt a more suited work-up and early treatment, to better predict prognosis, and to improve outcome of this subgroup of ischemic stroke patients.

## Material and methods

### Patients’ selection and clinical variables

The Acute STroke Registry and Analysis of Lausanne (ASTRAL) is a single center-based cohort registry of all acute ischemic stroke (AIS) admitted to the stroke unit and/or intensive care unit of the CHUV within 24 h of last well time, as published previously [15]. For this analysis, we included all consecutive patients from 2003 to 2016.

ASTRAL collects in a pre-specified manner a large range of parameters, which we analyzed retrospectively: demographics (age, sex, ethnicity), medical history and cardiovascular risk factors (pre-stroke modified Rankin score, previous stroke or transient ischemic attack or retinal ischemia, hypertension, diabetes mellitus and glucose intolerance, dyslipidemia, smoking, atrial fibrillation, symptomatic documented coronary artery disease, mechanical or biological valves, low ejection fraction < 35%, symptomatic peripheral artery disease, body mass index, oncological disease, migraine, alcohol abuse, and obstructive sleep apnea), and current medications (antiplatelets, anticoagulants, antihypertensives, lipid-lowering drugs, insulin and oral antidiabetics, hormones). The National Institute of Health Stroke Scale (NIHSS) score on admission is performed or supervised by NIHSS-certified personnel. Time from stroke onset to arrival at our institution is also recorded for each patient.

Comorbidities according to Elixhauser [16] and Charlson [17] indexes are collected and include the categories of congestive heart failure, cardiac arrhythmias, valvular disease, pulmonary circulation disorders, peripheral vascular disorders, hypertension, chronic paralysis and hemiplegia, dementia, other neurological disorders, chronic pulmonary disease, uncomplicated and complicated diabetes, hypothyroidism, renal failure, mild/moderate/severe liver disease, peptic ulcer disease, acquired immunodeficiency syndrome (AIDS), lymphoma, metastatic cancer, solid cancer, rheumatoid arthritis, coagulopathy, obesity, weight loss, fluid and electrolyte disorders, blood loss anemia, deficiency anemia, alcohol abuse, drug abuse, psychosis, depression, and myocardial infarction.

Vital signs (skin temperature, blood pressure, heart rate) and metabolic and hematologic parameters (glucose, creatinine, total cholesterol, white blood cells, hematocrit, and platelet count) are measured at admission (usually in the emergency room) and at 24–48 h after admission (usually in the stroke unit).

### Work-up and stroke management

Work-up of all AIS in our institution consists of acute brain imaging on admission, mainly based on computed tomography (CT)-imaging (16–detector row CT scanner until November 2005; 64–detector row CT scanner thereafter) and usually includes acute CT-angiography and CT-perfusion. Early ischemic signs on non-contrast CT (NCCT) are recorded, and ASPECTS (Alberta stroke program early CT score) calculated for strokes involving the MCA territory, and pc-ASPECTS [18] for patients with involvement of the posterior circulation.

We obtain at least one arterial study of cervical and cerebral arteries within 24 h of stroke onset, mainly CT-angiography (CTA) using multidetector-array technology in helicoidal mode, alternatively MR angiography (MRA) or Doppler including transcranial Doppler, or digital subtraction angiography (DSA) in patients considered for acute endovascular revascularization. On the first CTA or MRA (or Doppler, if none of them was performed), stenosis ≥ 50% and occlusion are recorded for all arterial segments and classified as intracranial or extracranial and inside or outside the ischemic territory.

Repeat brain CT or MRI is performed at approximately 24 h in patients receiving acute recanalization treatment. Imaging is also repeated (if possible, MRI) during hospital stay for any non-palliative patients when clinically indicated, such as a ≥ 2 NIHSS points worsening, with subsequent checking for ischemic and hemorrhagic changes; the latter are classified into clinically symptomatic hemorrhages according to ECASS-II [19]. In patients with initial arterial occlusion, recanalization are reassessed at 24 h (permitted range 12–48 h) using angio-CT, angio-MRI, or Doppler imaging.

The rest of the work-up for stroke mechanism also follows a pre-specified institutional protocol, which includes a minimum of 24-h cardio-respiratory and neurological monitoring in all patients. Transthoracic echocardiography is performed in all patients with unexplained embolic stroke and/or historical, clinical, or electrocardiographic clues for a cardiac source. Frequency and modalities of echocardiographic and angiographic imaging exams during the hospital phase are presented in the online supplementary Table 1.

Homocysteinemia is checked in patients below 55 years unless there is another clear cause of stroke.

There is no general consensus on when and how to perform a work-up for RMS [20]. In our institution, additional exams are performed if the above pre-specified work-up fails to identify an origin in young and elderly patients, especially if they have few risk factors, less atherosclerosis, stroke recurrences, and unexplained multifocal stroke. Unusual mechanisms are also sought in the presence of simultaneous ischemic and hemorrhagic strokes, with clinical clues pointing to a systemic disease, with a positive family history of unexplained cardiac or cerebrovascular disease, and if neuroimaging or biological tests showed unusual findings. Additional tests for such patients are targeted by a neurovascular specialist to the suspected cause and consist of one or several of the following: advanced arterial imaging (digital subtraction angiography, vessel wall-MRI), transesophageal echocardiography, prolonged portable monitoring after the hospitalization period, serological and cerebrospinal analysis for vasculitis, systemic immunological or neuroinfectious causes, fundoscopy and retinal angiography for inflammatory and genetic disorders, genetic testing for specific diseases (small vessel, intermediate vessel, large vessel dissections), anti-phospholipid antibodies (anti-cardiolipines, lupus anticoagulation, beta-2-glycoproteines, with repeat testing approximately 3 months later if initially positive), fibrin monomers, prostate-specific antigen, urological, gynecological evaluation, whole-body CT with contrast, and whole-body positron-emission tomography. Testing for coagulation factor deficiencies is only rarely performed because their association with arterial thrombosis remains uncertain [21].

Acute recanalization treatments, i.e., intravenous thrombolysis (IVT) and endovascular treatment (EVT), are used according to international and national guidelines at the time of admission. The rest of the acute stroke management and secondary prevention follow European Stroke Organization (ESO) guidelines at the time of hospitalization [22, 23].

### Determination of stroke mechanism and follow-up

Stroke mechanisms are classified in ASTRAL according to the TOAST trial [1] regarding small-vessel occlusion (lacunar) and atherosclerotic and cardioembolic mechanisms. Some of the cardioembolic definitions were adapted to reflect new scientific findings over the last 25 years. Furthermore, “multiple causes” were removed from the “undetermined etiology” category in TOAST in order to create a separate category. We have also added these frequent and well defined stroke mechanisms: dissection of supracardiac arteries [24], embolic stroke of undetermined source (ESUS) [25], patent foramen ovale (PFO) as a highly likely cause in patients with embolic stroke of undetermined cause and a RoPE score ≥ 7 [26], undetermined mechanism with incomplete workup, RMS, and multiple (simultaneous) causes. The precise definitions of the stroke mechanisms applied in ASTRAL are listed in the online supplementary Table 2.

For this study, we reviewed all strokes classified as RMS and as multiple (simultaneous) causes. Patients from the category “multiple mechanisms” were reclassified as RMS if one of the multiple causes was a rare cause. We then classified all RMS according to one of the following subtypes: vasculitis (primary systemic, secondary to systemic pathology, or isolated CNS vasculitis) or non-inflammatory vasculopathies, hematological disturbances, related to cancer (from either a hypercoagulable state or other mechanisms such as a direct mechanical effect), rare cardiac mechanisms, hemodynamic disturbances such as systemic hypotension, related to interventions, and monogenic diseases. Stroke were classified as caused by interventions if it was temporally related (i.e., usually within 24 h) to a diagnostic and/or therapeutic procedure. If such patients had their stroke while hospitalized, they were entered in ASTRAL if the stroke was the main clinical problem at some point during their hospitalization and if they were taken care of it in the stroke unit and/or the intensive care unit of our hospital.

Rankin-certified personnel assessed the Rankin score and living situation at 3 months in the outpatient clinic using the modified Rankin score (mRs). At 12 months, stroke recurrence and mortality were recorded in a structured telephone interview [27] by Rankin-certified personnel. If a recurrence was suspected, all available data (medical notes, discharge letters, radiology and radiology reports) were collected and reviewed by a vascular neurologist in order to ascertain the event. We acquired all outcome data.

### Study design and statistical analysis

AIS were divided in two groups: patients with a RMS and patients with all other mechanisms (OM).

To identify factors associated with a rare etiology, direct comparisons between all demographic, acute clinical, metabolic and radiological variables, and subacute radiological variables (symptomatic hemorrhagic transformation, recanalization) were first performed using a univariate analysis (UVA). The associations were expressed as odds ratios (OR) accompanied by 95% confidence intervals (CI). Bias from potentially missing dependent variables (RMS vs. OM) was avoided by excluding such patients rather than imputing this variable.

Of the 143 covariates available in the UVA, we selected the 81 for the multivariate analyses (MVAs) which were biologically most plausibly associated with RMS and who had only few missing values, regardless of their significance. All analyses were performed using the binomial model or the proportional odds approach, depending on the number of categories of the response variable. This approach takes into consideration multiple interrelationships, and removes covariates considered to convey similar biological information, as described by Harrell [28]. In all MVAs, imputation of missing independent covariates was carried out using multiple chain equations methodology [29]*.* In this way, we generated five complete datasets. Analysis of each dataset was performed separately, and results of these analyses were appropriately combined to generate final conclusions.

Handicap at 3 months was assessed with a Rankin-shift analysis. A proportional odds model was employed in which the mRS (dependent variable) had originally 6 levels, but levels 5 and 6 were collapsed into a single category while levels 0–4 were retained as distinct. In this model, the treatment odds ratios between a specific level and its next in ascending order are assumed constant, so a single parameter (common OR) summarizes the shift in outcome distribution between cases and controls. Again, all variables from the UVA were used for the MVAs, independently of their significance.

Stroke recurrence and mortality over the first 12 months were analyzed using a proportional odds model, as described previously.

Statistical analysis was performed with R statistical software (version 3.6.3) software, while level of significance was set at 5% throughout.

### Data availability statement

An anonymized copy of the data used for the current project can be obtained by writing to the last author. Data will be provided based on a reasonable request describing its reasons, methods of planned analysis, and type and authorship of a potential publication, if applicable.

## Results

### Patients’ characteristics

Four thousand one hundred fifty-four AIS were registered in ASTRAL between 2003 and 2016 of which 222 were classified as RMS (5.3%). In this group, 42.0% were female and median age was 66 years (IQR 20.8). Stroke mechanisms was determined in all patient (no missing dependent variables). Among the 222 RMS, 27 (12%) had multiple mechanisms of stroke; as mentioned above, these were considered RMS for the current analysis. Etiologies of this group are detailed in online supplementary Table 3. When comparing pure RMS with RMS and OMS, demographics, stroke severity, and outcomes were similar except for younger age in pure RMS (see online supplementary Table 4).

Further clinical characteristics of the studied patients are described in Table 1 and online supplementary Table 5, whereas radiological and laboratory differences are listed in the supplementary Table 6.

**Table 1:** Clinical characteristics of the overall population of patients with RMS and OM (control group). An asterisk highlights the statistically significant differences seen in the univariate comparison

Variables	Overall population included (*N* = 4154)	Rare stroke mechanisms (*N* = 222)	All other mechanisms (*N* = 3932)	OR (95% CI)	*p* value
**Demographic variables**					
Age (years)	73.6 (20.8)	66.0 (20.8)	74.0 (20.4)	0.97* (0.96–0.97)	< 0.01
Sex (females)	1841/4154 (44.3%)	94/222 (42.3%)	1747/3932 (44.4%)	0.92 (0.70–1.21)	0.542
NIHSS	6	7	6	1.01 (0.99–1.03)	0.339
**Clinical variables**					
Time to arrival at hospital (hours)	3.2 (8.9)	2.2 (6.9)	3.3 (9.0)	0.96* (0.94–0.99)	< 0.01
Bilateral stroke localization	394/4137 (9.5%)	43/220 (19.5%)	351/3917 (9.0%)	2.47* (1.66–3.63)	< 0.01
Acute revascularization treatment (IVT and/or EVT)	929/4153 (22.4%)	30/221 (13.6%)	899/3932 (22.9%)	0.54* (0.36–0.79)	0.016
**Stroke-risk factors**					
Hypertension	2973/4153 (71.6%)	114/221 (51.6%)	2859/3932 (72.7%)	0.40* (0.30–0.53)	< 0.0.1
Diabetes	766/4150 (18.5%)	26/222 (11.7%)	740/3928 (18.8%)	0.57* (0.37–0.85)	< 0.01
Cholesterol	3048/4149 (73.5%)	130/220 (59.1%)	2918/3929 (74.3%)	0.50* (0.38–0.66)	< 0.01
Smoking	949/4110 (23.1%)	72/220 (32.7%)	877/3890 (22.5%)	1.67* (1.24–2.23)	< 0.01
Atrial fibrillation	1241/4148 (29.9%)	21/222 (9.5%)	1220/3926 (31.1%)	0.23* (0.14–0.36)	< 0.01

RMS were similarly frequent in patients from the primary catchment area from our stroke center and in referred patients (5.2% among the 3520 primary catchment patients (85%) vs. 6.8% among the 766 referred patients (15.0%), (*p* = 0.48). There was also no difference between RMS and OM originating from the primary catchment area (RMS 173/222 (78.0%) vs. OM 3347/4154 (83.0%)) and the referral area (RMS 49/222 (22.0%) vs. OM 717/4154 (17%), (*p* = 0.48).

### Rare stroke mechanisms

Stroke mechanisms within the RMS group were linked to vasculitis (11.7%) or non-inflammatory vasculopathies (10.8%), hematological abnormalities (6.3%), related to cancer (22.5%), rare cardiac mechanisms (10.8%), hemodynamic disturbances such as systemic hypotension (3.6%), related to diagnostic and therapeutic interventions (25.6%), and associated with genetic diseases (2.2%), as described in more detail in the Table 2.

**Table 2:** Etiological classification of AIS presenting as RMS, presented as categories and sub-groups (*N* = 222)

Vasculitis	26
Primary systemic	9
Temporal arteritis (7), polyarteritis nodosa (1), Churg-Strauss syndrome (1)	
Associated with systemic diseases	5
Behçet’s disease (2), sytemic erythematosus lupus (2), Wiscott-Aldrich syndrome (1)	
Associated with infections (central nervous or systemic)	3
Systemic varicella-zoster virus (1), cerebral toxoplasmosis (1), neuroborreliosis (1)	
Isolated vasculitis of the central nervous system	9
**Hypercoagulable state**	**14**
Hyperhomocysteinemia	3
Genetic (2), malnutritional (1)	
Anti-phospholipid antibodies syndrome	9
Non neoplastic disorders of blood viscosity	2
Severe iron deficiency (1), cryoglobulinemia from hepatitis C (1)	
**Neoplasm related**	**51**
Solid cancer	35
Lung (7), prostate (4), breast (4), gallbladder (3), pancreas, bladder, uterus, gastric, esophagus (2 each), hepatic, vulvar, sarcoma, seminoma, intestinal, ovary, generalized-undetermined (1 each)	
Hematological neoplasia	8
Polycythemia vera (4), leukemia (2), multiple myeloma (2)	
Mechanical effect of tumor	2
Cerebral germinoma (1), cerebral astrocytoma (1)	
Late effect of chimio and/or radiotherapy	6
**Drug related**	**6**
Drug abuse	5
Cocaine (without vasospasms) (3), cocaine and heroin sniffing (without vasospasms) (1), 3,4-methylenedioxy-methamphetamine (1)	
Medication related	1
Clozapine-induced myocarditis with intracardiac thrombus (1)	
**Migrainous stroke**	**1**
**Vasospasm**	**6**
Reversible cerebral vasoconstriction syndrome	4
Induced by cocaine	2
**Rare cardiac causes**	**24**
Endocarditis	21
Cardiac tumor	3
Sarcoma (2), myxoma (1)	
**Related to medical intervention**	**57**
Diagnostic procedures	29
Coronarography (21), arterial/cardiac catheterization (3), venous catheterization leading to paradoxical embolization through a PFO (2), cerebral angiography (2), carotid sinus massage (1)	
Valve surgery/replacement	6
Biological (4), mechanical (1), endovascular valve dilatation (1)	
Coronary/cardiac surgery	3
Aortic surgery	6
Carotid surgery	7
Cervical/cerebral artery endovascular revascularization	3
Gas emboli	3
Dialysis (1), transthoracic pulmonary biopsy (1), peripheral venous catheterization (1)	
**Genetic diseases**	**5**
Genetic collagen disorders	3
Rendu-Osler-Weber disease (2), Marfan’s syndrome (1)	
CADASIL (cerebral autosomic dominant arteropathy with subcortical infarcts and leucoencephalopathy)	2
**Other non-inflammatory vasculopathies**	**24**
Moyamoya disease	3
Cerebral amyloid angiopathy with ischemic manifestations	1
Direct contact of cervical arteries with bone, leading to embolization	2
Aneurysms with ischemic manifestations	17
Basilar artery (12), vertebral artery (1), medial cerebral artery (4)	
Post-infectious	1
**Hemodynamic**	**8**

In the control population with OM, stroke mechanisms were cardioembolic in 1307 patients (33.0%), atherosclerotic in 631 (16.0%), ESUS in 516 (13%), small vessel occlusion in 489 (12.0%), undetermined with incomplete workup in 444 (11.0%), dissection in 171 (4.0%), multiple/coexisting non-rare mechanism in 226 (5.7%), and PFO in 148 (3.7%).

### Independent predictors of rare stroke mechanisms

In the multivariate analysis, we identified multiple demographic, clinical, laboratory, and radiological factors that were independently associated with RMS (Table 3). These included younger age, earlier hospital arrival, simultaneous bilateral stroke localization, a history of previous ischemic stroke or transient ischemic attack, pre-treatment with lipid-lowering agents, certain comorbidities such as cardiac valvular pathology, coagulopathy, oncological disease, depression, drug abuse, and AIDS. In this last group, HIV infection was known in all before the index stroke. Common cardiovascular risk factors such as hypertension, diabetes, and atrial fibrillation were less frequent in RMS.

**Table 3:** Significant results of the multivariate comparison of baseline demographic, clinical, radiological and laboratory variables between RMS and OM

Variables	OR	95% CI	*p* value
**Demographic and clinical variables**			
Age (per year)	0.97	0.96–0.98	< 0.01
Time to arrival at hospital (per hour)	0.95	0.93–0.98	< 0.01
Bilateral stroke localization	2.09	1.39–3.14	< 0.01
**Risk factors and comorbidities**			
Previous ischemic stroke or TIA	1.48	1.06–2.07	0.022
Lipid-lowering medication before onset	1.56	1.08–2.25	0.017
Hypertension	0.68	0.47–0.98	0.04
Diabetes	0.61	0.38–0.97	0.037
Atrial fibrillation	0.27	0.16–0.45	< 0.01
Any valvular pathology	1.58	1.10–2.28	0.014
Acquired immune deficiency syndrome	8.24	1.65–41.15	0.023
Deficiency anemia	2.12	1.29–3.47	< 0.01
Coagulopathy	2.54	1.48–4.36	< 0.01
Solid cancer	2.48	1.65–3.73	< 0.01
Metastatic cancer	5.82	3.25–10.42	< 0.01
Drug abuse	2.69	1.35–5.39	< 0.01
Depression	2.07	1.22–3.51	< 0.01
**Physiological and laboratory variables**			
Higher temperature	1.30	1.01–1.67	0.045
Higher diastolic blood pressure	0.89	0.81–0.98	0.023
Hemoglobin	0.98	0.97–0.99	< 0.01
Platelets	0.98	0.96–1.00	0.016
**Radiological variables and acute treatment**			
Early ischemic changes on CT scan	1.71	1.14–2.56	0.01
Extracranial pathology on CTA	0.42	0.23–0.76	< 0.01
Any acute revascularization treatment	0.46	0.30–0.69	< 0.01
Symptomatic hemorrhagic transformation on subacute imaging	4.67	2.19–9.97	< 0.01

Among biological variables, increased admission temperature, lower acute blood pressure, and lower hemoglobin and platelet levels were also associated with RMS. Radiological and treatment variables significantly linked to a higher risk of RMS were early ischemic changes on NCCT at admission, less extracranial arterial pathology, and presence of a symptomatic hemorrhagic transformation on the 24-h neuroimaging, independently of revascularization treatment. Finally, RMS patients were treated only half as often with IVT and/or EVT.

### Outcome of AIS in RMS

**Fig. 1:**
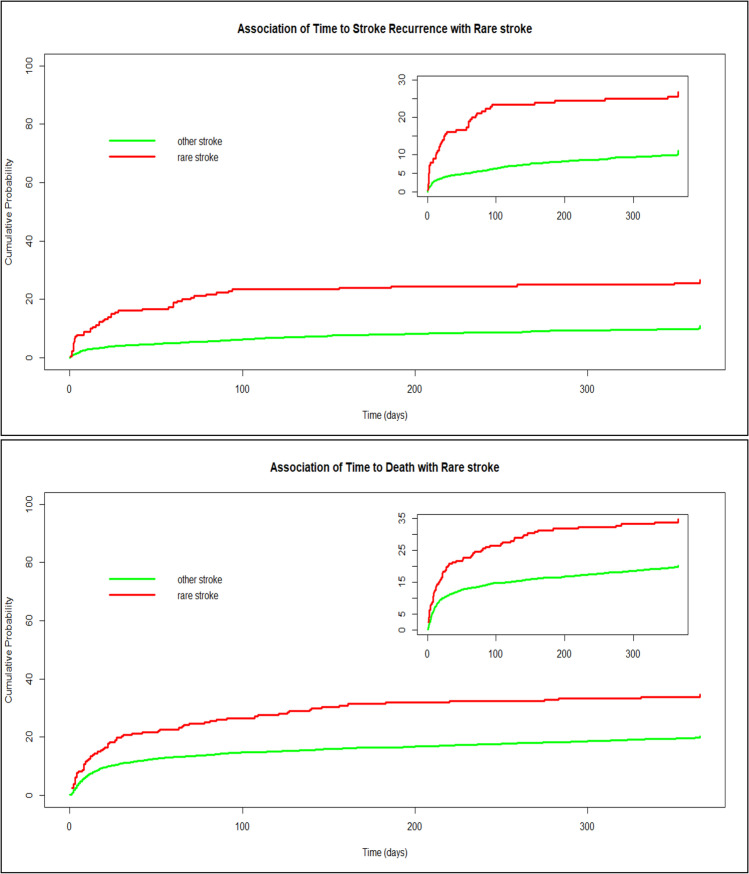
Recurrence and mortality. Recurrence (upper graph) and survival (lower graph) plots over 12 months, censored for recurrence or drop-out, scaled from 0 to 100% (insert: scaled from 0 to 30%)

RMS patients had a significantly worse outcome for several measures in unadjusted analysis (Table 4 and Fig. 1). When considering each specific sub-groups of rare mechanisms, this was mainly explained by the “neoplasm related” and the “rare cardiac” categories (Table 5).

**Table 4:** Unadjusted long-term outcomes and stroke-recurrence rates. All differences are statistically significant

Variable	Overall population	RMS	OM	Un-adjusted OR	OR-CI	*p* value
*N*	4154	222	3932			
Functional outcome at 3 months (median mRs)	2.0 (3.0)	2.0 (5.0)	2.0 (3.0)	1.16	1.08–1.24	< 0.01
Favorable outcome at 3 months (mRs 0–2) (*N*, %)	2355/3950 (59.6%)	113/216 (52.3%)	2242/3734 (60.0%)	0.73	0.55–0.96	0.025
Mortality at 12 months (*N*, %)	800/3794 (21.1%)	72/207 (34.8%)	728/3587 (20.3%)	2.09	1.55–2.81	< 0.01
Stroke recurrence at 12 months (*N*, %)	335/3614 (9.3%)	48/210 (22.9%)	383/3824 (10.0%)	2.90	2.04–4.05	< 0.01

**Table 5:** Unadjusted short-term outcomes for each etiology subgroup. Statistically significant results are highlighted with an asterisc

Etiology sub-group	*n*	mRs 0 − 2	mRs 3 − 6	OR	*p* value
Vasculitis	26	16 (62%)	10 (38%)	1.05 (0.47–2.33)	0.892
Hypercoagulable state	14	10 (71%)	4 (29%)	1.65 (0.51–5.27)	0.397
Neoplasm related	52	19 (37%)	33 (63%)	0.38* (0.21–0.67)	< 0.01
Drug related	6	5 (83%)	1 (17%)	3.30 (0.38–28.28)	0.275
Migrainous	1	1	0	–-	–-
Vasospasm	6	3 (50%)	3 (50%)	0.66 (0.13–3.27)	0.611
Rare cardiac	24	9 (37%)	15 (63%)	0.39* (0.17–0.90)	0.028
Medical interventions	52	26 (50%)	26 (50%)	0.66 (0.38–1.14)	0.137
Genetic	5	4 (80%)	1 (20%)	2.64 (0.29–23.64)	0.385
Vasculopathy	24	14 (58%)	10 (42%)	0.92 (0.40–2.08)	0.845
Hemodynamic	8	6 (75%)	2 (25%)	1.98 (0.39–9.82)	0.403

**Fig. 2:**
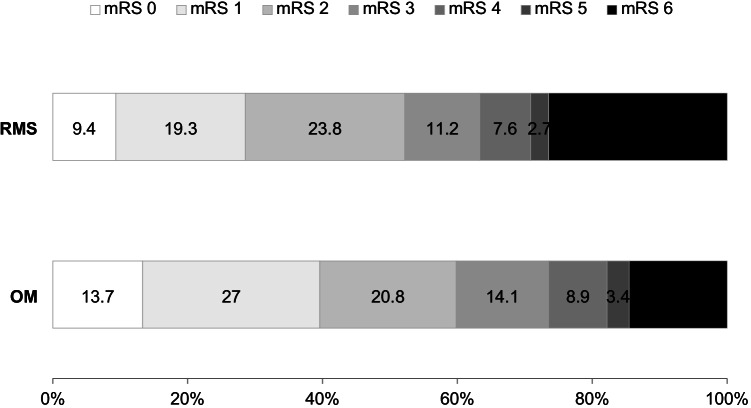
Comparison of functional outcome (mRs) between RMS and OM groups

In adjusted analysis, functional outcome at 3 months remained clearly less favorable (OR_adj_ for Rankin shift: 1.74, 95% CI 1.25 – 2.43). A 12-month mortality was increased (OR_adj_ 2.41, 95% CI 1.48 – 3.94) (see also Fig. 2A for survival curve), as was the likelihood of stroke recurrences over the first 12 months (OR_adj_ 1.99, 95% CI 1.26 – 3.15) (see also Fig. 2B for free-of -recurrence curve).

## Discussion

In this large cohort of consecutive AISs from a comprehensive stroke center, RMS accounted for 5.3% of all AIS, with similar rates in the primary catchment and referred patients. Among the rare mechanisms, the most represented categories were related to medical interventions, followed by cancer, vasculitis, rare cardiac mechanisms, and hematological disturbances. Of note, genetic causes and migrainous stroke were exceedingly rare. Predictive factors linked independently to RMS were younger age, bilateral localization of stroke, previous cerebrovascular events, absence of traditional vascular risk factors, and several comorbidities (such as cardiac valvular pathology, coagulopathies, cancer, drug abuse, and AIDS). In addition, several biological and radiological features distinguished these patients. Despite lower recanalization treatments, symptomatic hemorrhagic transformations were more frequent in RMS, as were poor functional outcome, mortality, and stroke recurrences in the long term. Interestingly, we found that lipid-lowering pre-stroke treatment and pre-existing cardiac valvular pathology were associated with a higher likelihood of RMS. This can best be explained by the RMS subgroup “related to medical intervention” with a significant number of patients with cardiac interventions [30] with valvular disease and pretreatment with lipid-lowering medication. Higher pre-stroke depression in RMS may be explained by more frequent comorbidities like cancer and other systemic diseases, often associated with emotional problems [31]. We did not find a good explanation why ischemic changes on admission CT were associated with RMS independently of stroke severity and delay to arrival.

The frequency of RMS in our cohort was similar to that of previous studies [11–13]. As with the only other smaller study on RMS using adjusted analyses [14], we found RMS to have lower age, less traditional cardiovascular risk factors, and a similar gender distribution. However, in contrast to that study, we identified a high prevalence of RMS linked to medical interventions, cancer, and vasculitis, and a lower prevalence of hypercoagulable states and infections. These differences could perhaps be explained by the different stroke populations and a higher sensitivity, availability, and use of diagnostic tools for detecting RMS in our cohort, which is about 10 years more recent than the previous study. It should also be noted that this previous study did not include all types of cancer but just hematologic malignancies, which may have a significant influence on the prognosis of the cohort.

On radiological examinations, RMS patients had a significantly higher frequency of early ischemic changes on acute imaging (mostly CT-based), but less extracranial vascular pathology, which is consistent with the lower rate of traditional cardiovascular risk factors in this group.

RMS patients were less likely to receive revascularization treatment (IVT and/or EVT). This may be due to a higher frequency of unusual presentations (type of onset, symptoms) in younger patients, resulting more often in misdiagnosis [32]. Furthermore, the strokes related to medical interventions are often detected late after waking up from general anesthesia. Finally, some rare etiologies (like vasculitis, endocarditis, or paraneoplastic syndrome in patients with active cancer) contra-indicate the administration of IVT. Despite this lower rate of revascularization treatments, we found a significantly higher rate of hemorrhagic transformation of the ischemic stroke in patients with RMS. This finding might be explained by the fact that more malignant conditions with direct effect on brain vessels (vasculitis) [33] and a higher bleeding risk (endocarditis, coagulopathy) [34, 35] were present in the RMS group. This higher bleeding risk may have direct implications if considering patients with RMS for IVT.

RMS patients had both a higher frequency of preceding and subsequent vascular events, pointing to the difficulties of preventing and treating such stroke mechanisms. Regarding the further evolution, we suspect that their significantly poorer long-term outcome and higher mortality could also be due to the limited treatment options, as indicated by the significant worse outcome for AIS related to neoplasm and rare cardiac mechanism. Further aggravating factors may be the higher load of specific comorbidities associated with poorer outcome (cancer, drug abuse, AIDS, depression), and the later identification of the (rare) stroke mechanism.

Our description of an RMS-patient profile has direct clinical implications: the possibility of a RMS should be raised in younger patients with few traditional risk factors, unexplained stroke recurrences, absence of significant arterial pathology, and with certain comorbidities including cancer, drug abuse, AIDS, and coagulopathy. With earlier identification of RMS, more rapid and targeted treatment could possibly improve patient outcomes and reduce mortality rates. Still, the prognostic accuracy could be improved, and patients with RMS and their relatives should probably be informed of the higher risk of stroke recurrence and poorer outcome.

To the best of our knowledge, this is the largest case-controlled study focusing on RMS with a comprehensive classification and an extensive dataset analysis. This allowed us to identify 24 independent factors associated with RMS. Other strengths of our work include the consecutive nature of the collected data over a long period of time, with pre-specified and standardized data collection using up-to-date scales, definitions and neurovascular imaging methods, and a population typical of stroke centers that include both primary and tertiary-referred patients.

The limitations of our analysis are its retrospective, observational, and non-randomized nature based on a single stroke-center rather than a population. Our study population consists mainly of a typical Western European community of elderly Caucasian patients. Hence, our results need to be confirmed in other ethnicities, regions, and socioeconomic settings. Our registry is also limited to patients admitted within 24 h of stroke onset; although RMS patients arrived significantly earlier at the hospital in this time window than non-RMS patients, some RMS patients may present later than 24 h after stroke onset, making our sample not necessarily representative of all RMS types. Our study population also contains 6% in-hospital strokes, and such patients may have different comorbidities and other features than out-of-hospital strokes. Similarly, patients with periprocedural mechanisms may dilute other associations of RMS with patient characteristics. Although our stroke work-up is standardized, we did not perform a search of RMS in every patient, in particular not in patients with an obvious alternative cause. This reflects the general uncertainty about the indication and extent of additional exams required in patients without an obvious cause in the standard work-up [20]. Therefore, some RMS may have been underrecognized. Finally, we have structured the classification of RMS in the most comprehensive way possible, but these categories are still somewhat arbitrary and therefore debatable.

## Conclusion

RMS affect about one in 20 AIS patients and are a heterogeneous group of etiologies, associated with higher recurrence rates and worse outcome. Our findings that RMS are more frequent in patients with few traditional risk factors and with specific systemic comorbidities may allow more rapid investigations and identification of such patients. This could lead to faster and better treatment, and more favorable outcomes in the long term.

## Supplementary information

Below is the link to the electronic supplementary material.

Supplementary file1 (DOCX 61 KB)
