# Supplementary material for: Rare stroke mechanisms in 4154 consecutive patients: causes, predictors, treatment, and outcomes
Source: Neurol Sci. 2022 Aug 22;43(11):6359–69. doi: 10.1007/s10072-022-06344-w (PMC9616760; doi:10.1007/s10072-022-06344-w)
Supplement: Supplementary file 1 — Supplementary file1 (DOCX 61 KB) [file 10072_2022_6344_MOESM1_ESM.docx]

**Rare stroke mechanisms in 4154 consecutive patients: causes, predictors, treatment and outcomes**

**Online supplementary tables and figures**

**Online supplementary table 1:** Diagnostic work-up

**Online supplementary table 2:** Classification of stroke mechanism in ASTRAL

**Online supplementary table 3:** Subgroup RMS&OM etiologies

**Online supplementary table 4:** Univariate comparison of the mixed etiology (RMS&OM) and “pure” RMS subgroups.

**Online supplementary table 5:** Additional demographic, clinical, and risk factor data in the study population

**Online supplementary table 6:** Acute physiological, radiological and laboratory features in the study population

**Online supplementary table 1:** Diagnostic work-up

| **Echocardiography** | **Total (n=4141)** |
| --- | --- |
| Transthoracic (TTE) only | 2140 (52%) |
| Not done or other | 1235 (30%) |
| Assessment before hospital admission for stroke | 329 (8%) |
| Transesophageal (TEE) and TTE | 319 (8%) |
| Planned just after hospitalization | 91 (2%) |
| TTE only | 27 (<1%) |
| **Acute/subacute angiography imaging** | **Total (n=3731)** |
| Acute CT angiography (CTA) | 2814 (68%) |
| Acute CTA & neurosonology | 626 (15%) |
| None or other | 504 (12%) |
| Acute MR angiography (MRA) | 59 (1%) |
| Neurosonology | 60 (1%) |
| Acute CTA & MRA | 39 (1%) |
| Acute CTA & MRA & neurosonology | 22 (<1%) |
| Acute MRA & neurosonology | 17 (<1%) |

Data on echocardiography are missing for 13 patients and data for angiography are missing for 423 patients. Neurosonology stands for cervical Doppler/vascular ultrasound, with added transcranial Doppler whenever technically feasible (good bone window).

**Online supplementary table 2**. Classification of stroke mechanisms in ASTRAL

# Classification of stroke mechanisms as used in the Acute STroke Registry and Analysis of Lausanne (ASTRAL)

Main adaptations from the classical TOAST classification (1) : the ASTRAL classification

- keeps the essentials of atherosclerotic, cardioembolic, and small vessel occlusion definitions from TOAST
- adds the categories „Dissection“, „PFO as most likely cause“, “embolic stroke of undetermined etiology (ESUS)”, and “multiple/coexisting causes”
- reclassifies some cardiac sources into “high”, “low-medium” or “rare stroke” categories according to new knowledge since the original TOAST classification
- removes the „multiple causes” from the “undetermined etiology”

**1. Large-artery atherosclerosis**

Stenosis ≥ 50% or occlusion of a major artery or branch cortical artery leading to the ischemic territory which is presumably due to atherosclerosis, or a mobile thrombus = floating thrombus (presumably) attached to an atherosclerotic plaque in the aortic arch (up to 2cm distal to the left subclavian because of frequent diastolic flow reversal,(2), cervical or intracranial arteries .

Imaging : if visible, infarct > 15 mm (or smaller but superficial localization).

If these criteria are met for at least one acute lesion, but there are multiterritory lesions, the classification is still allowed (assuming that A) a carotid stenosis may also produce contralateral ACA or junctional lesions, or B) that activation of several plaques has occurred through a systemic/inflammatory process).

**2. Cardioembolic stroke**

Patients with ≥1 high risk cardiac source as listed below (medium risk sources: classified as “possible” in original TOAST paper (1) 🡪 not classified as “cardiac” in ASTRAL but as “undetermined”).

Other specified causes (such as dissection, stroke of other determined origin) should be eliminated.

#### Cardiac conditions considered the cause of stroke and TIA according to Adams 1993

#### Modified according to (3-8)

| **High risk sources for recurrent stroke** | **Low-medium risk sources for recurrent stroke** |
| --- | --- |
| Atrial fibrillation (chronic, paroxysmal or persistent) with or without valvular disease, even if fully anticoagulated | Mitral valve prolapse (especially with advancing age) Aortic valve stenosis |
| Atrial flutter | Myocardial infarction > 4 weeks and < 6 months |
| Recent (transmural) myocardial infarction (< 4 weeks) | Hypokinetic left ventricular segment  (dyskinesia without aneurysm: not source) |
| Akinetic left ventricular segment; left ventricular aneurysm | Moderate/severe mitral stenosis without atrial fibrillation |
| Left atrial or ventricular thrombus | Bioprosthetic cardiac valve including TAVI |
| Mechanical prosthetic valve (even if fully anticoagulated) | Congestive heart failure (clinical syndrome) |
| Dilated cardiomyopathy with ejection fraction < 35% | Left atrial turbulence (smoke) |
|  | Sick sinus syndrome |
|  | Mitral annulus calcification |

## Note: as opposed to the original TOAST classification, the following causes of cardiac stroke were classified in ASTRAL under

## “Other determined origin / rare causes (rare cardiac causes)”: infective endocarditis, atrial myxoma and other left intracardiac tumours, cardiac/valvular interventions, and nonbacterial thrombotic endocarditis

## “Likely PFO/ASD-related”: PFO/ASIA/ASD with specific criteria (see below)

## Low-medium risk sources were not considered as sufficient to classify a stroke as “cardioembolic”.

**3. Small vessel disease (SVD)**

Clinical syndrome compatible with a small deep infarct and

- No lesion on imaging,
- Or MRI or CT showing one or several (multiple) acute lacunar infarction(s) (according to ASCOD) (5), i.e. one or several small deep infarct < 15mm corresponding to the symptoms

and ≥1 of the following:

A) Old small deep infarct(s) of lacunar type in other territory(ies)

B) “Severe” leukoaraiosis, or microbleeds or severe dilatation of perivascular spaces.

C) Hypertension, or age >65, or ≥1 CVRF of any type

If none of the criteria A-C are fulfilled, this is usually a “11” (Undetermined other, non-ESUS), or “10” (Undetermined causes non-SVD, incomplete work-up)

If *simultaneous* criteria for SVD and for atherosclerotic (1), cardioembolic (3), dissection (5) or other determined etiology (6) are fulfilled, the stroke will be classified as follows:

- if the above criteria for a *SVD* are fulfilled 🡪 “Multiple/coexisting”
- if imaging SVD negative or criteria for lacunar infaction *not* fulfilled (i.e. no A) or B) 🡪 classify for the other cause.

***Cave***: in the classical TOAST classification (1)

- Small vessel occlusion (lacune) was defined as “traditional lacunar syndrome without evidence of cerebral cortical dysfunction
  - no radiological lesion
  - or relevant subcortical or brainstem lesion <15 mm
  - diagnosis supported by hypertension or diabetes
- This category cannot co-exist with cardiac/athero

**4. Dissection**

At least one of the following radiological features has to be present in an intra- or extracranial artery (including aortic arch) in relation with the ischemic territory on any type of arterial imaging (9) : mural hematoma, long filiform stenosis, pseudo-aneurysm, double lumen, intimal flap, and occlusion situated more than 2 cm above the bifurcation of the carotid artery revealing a dissecting aneurysm or a long filiform stenosis after recanalisation.

## 5. Other determined origin / rare causes

These are rare, defined causes of stroke.

They are separated in distinct categories, which are described here:

- Non-infectious vasculitis, i.e. primary systemic vasculitis, vasculitis associated with systemic diseases, or isolated vasculitis of the CNS (10)
- Hypercoagulable states other than neoplasm-related, such as hyperhomocysteinemia, anti-phospholipid antibodies syndrome, non-neoplastic disorders of blood viscosity (f.ex. cryoglobulinemia or severe iron deficiency))
- Related to neoplasms, oncological diagnostics, or oncological treatments
- Cancer-related
  - Cancer-related coagulopathy: non-bacterial thrombotic endocarditis, demonstrated on echocardiography with vegetations in a patient not meeting criteria for infective endocarditis, and/or ≥2 of the following
    - Recurrent unexplained arterial and/or venous thrombotic events while pat has active cancer (independently whether PFO present)
    - Radiologically multifocal non-lacunar (> 1 territory) stroke lesions, either simultaneous, or acute & subacute (not chronic)
    - Blood markers of hypercoagulability: ≥1 of the following : D-dimers level > 4 μg/mL on admission, and/or presence of monomers at any stage during the current stroke-related hospitalization, and/or thrombocytosis > 1’000 G/L, and/or hyperleukocytosis > 100 G/L
  - Direct tumor invasion of blood vessels, i.e. tumor embolization, leptomeningeal carcinomatosis, brain intravascular lymphoma, or direct vessel compression/infiltration by tumor
  - Cancer-related immunosuppression leading to systemic or CNS infection leading to stroke (such as infectious endocarditis, bacterial meningitis etc.)
- Oncological diagnostic procedure-related, i.e. stroke related to surgical or endoscopic diagnostic biopsy
- Cancer therapy-related: ≥1 of the following
  - Prothrombotic chemotherapies and hormonal therapies (ref: Marto et al. Drugs associated with ischemic stroke: Stroke, 2021; 52(10): e646–59
  - Cancer-therapy related immunosuppression leading to systemic or CNS infection leading to stroke
  - Cardiac failure with ejection fraction <35% attributed to past chemotherapy and/or radioltherapy
  - Past radiotherapy: remote (> 1 year, independently of dose) radiation
    - to head/neck/mediastinum with demonstrated large artery arteriopathy leading to the ischemic territory (≥50% stenosis, and/or high risk plaque radiology) insufficiently explained by ≥2 vascular risk factors
    - to head, with small vessel cerebral arterial pathology not sufficiently explained by ≥2 vascular risk factors
  - Oncological therapeutic procedures/surgeries/interventions
- Non-inflammatory vasculopathies and vascular malformations, such as fibromuscular dysplasia, embolic from aneurysms, neurofibromatosis-related vasculopathy
- Hemodynamic disturbances, such as systemic hypotension
- Vasospasm, either idiopathic or secondary to other diseases or substances
- Pregnancy and delivery related, such as post-partum angiopathy or amnionic fluid embolism
- Migrainous stroke, as defined by the International Headache Society (11)
- Rare cardiac mechanism, such as myxoma, fibroelastoma,
- Related to interventions : diagnostic : temporally related (i.e. usually within 24 hours) to a diagnostic procedure
- Related to interventions : diagnostic : temporally related (i.e. usually within 24 hours) to a therapeutic procedure
- Genetic, i.e. monogenic diseases such as CADASIL, Fabry’s disease
- Infectious/parainfectious, such as neurosyphilis, likely caused by influenza or SARS-Co2,
- Other rare causes

**6. Undetermined causes ESUS, non-SVD, complete work-up (embolic lesion shown)**

ESUS = embolic strokes of unknown origin according to (6)

- Stroke detected radiologically (CT or CTP or MRI) that is not lacunar (lacunar= subcortical ≤15mm (≤20mm on DWI) in largest dimension, including on DWI, in the distribution of the small penetrating cerebral arteries (visualisation by CT usually needs delayed imaging > 24-48h after stroke onset))
- Absence of extracranial or intracranial atherosclerosis causing ≥50% luminal stenosis in arteries supplying the area of ischaemia
- No major-risk cardioembolic source of embolism (permanent or parosysmal AF, sustained atrial flutter, intracardiac thrombus, prosthetic cardiac valve, atrial myxoma or other cardiac tumours, mitral stenosis, recent (<4 weeks) myocardial infarction, left ventricular ejection fraction < 30%, valvular vegetations, or infective endocarditis
- No other specific cause of stroke (e.g., arteritis, dissection, migraine/vasospasm, drug misuse)
- Complete work-up (i.e. including arterial imaging, echocardiography, and ≥24h continuous arrhythmia monitoring),
- Do not fulfill any of the other criteria for an etiology of the ASTRAL classification.

These ESUS patients may have findings that are considered incidental (valvular strands, mitral valve prolapse, mitral annulus calcification, unstable non-stenosing plaques, plaques in the aortic arch < 4 mm in thickness and without mobile component).

A floating thrombus (stenosing or not) without a clear underlying cause (plaque, cardiac, dissection, post-actinic, hematological etc.) and an embolic lesion is also classified as ESUS.

Note: ESUS in a patient with PFO and RoPE score ≥7 are classified “Likely PFO/ASD-related” in ASTRAL.

Note: if a patient has several small deep infarcts < 15mm fulfilling SVD-criteria, they are considered SVD (not “embolic”/ESUS).

**7. Coexisting and multiple causes**

Patients fulfilling the criteria of at least two of the following causes: 1, 3, 4 or 5, 6.

**8. Likely patent foramen ovale (PFO) and/or atrial septum defect (ASD) related**

Calculate the RoPE score for all patients with PFO and/or ASD.

Classify as PFO/ASD

- if RoPE score ≥ 7,
- or any RoPE-score, but simultaneous/recent DVT and/or PE
- or any RoPE-score, but documented PFO-thrombus.

If RoPE score < 7, classified as

- 7= Undetermined, complete work-up, ESUS
- or: 10= Undetermined, incomplete work-up
- or: 11= Undetermined other (lacunar non-SVD; imaging neg. non-SVD; etc. ....), complete work-up

If criteria for another cause are fulfilled and a simultaneous PFO/ASD with RoPE score ≥ 7 is present: classify for the other cause (not “multiple”).

Depending on the research question, patient classified as PFO/ASD stroke

- may be considered “ESUS” if an embolic lesion has been documented
- may be grouped with undetermined=cryptogenic causes

**9. Undetermined causes non-SVD, incomplete work-up**

Other strokes not fulfilling the SVD criteria, with or without a radiological lesion, but without complete work-up, i.e. typically missing at least one of the following:

- arterial imaging of intracranial and/or cervical arteries
- echocardiography
- ≥24h continuous arrhythmia monitoring

**10. Undetermined other, non-ESUS, complete work-up**

Other strokes with complete work-up not fulfilling other criteria, i.e. typically

- absence of a radiological embolic lesion but not corresponding to SVD
- lacunar lesion but not other SVD criteria

**Bibliography**

1. Adams HP, Jr., Bendixen BH, Kappelle LJ, Biller J, Love BB, Gordon DL, et al. Classification of subtype of acute ischemic stroke. Definitions for use in a multicenter clinical trial. TOAST. Trial of Org 10172 in Acute Stroke Treatment. Stroke. 1993;24(1):35-41.

2. Harloff A, Simon J, Brendecke S, Assefa D, Helbing T, Frydrychowicz A, et al. Complex plaques in the proximal descending aorta: an underestimated embolic source of stroke. Stroke. 2010;41(6):1145-50.

3. <https://ccs.mgh.harvard.edu/ccs_title.php> [

4. Ay H, Furie KL, Singhal A, Smith WS, Sorensen AG, Koroshetz WJ. An evidence-based causative classification system for acute ischemic stroke. Ann Neurol. 2005;58(5):688-97.

5. Amarenco P, Bogousslavsky J, Caplan LR, Donnan GA, Hennerici MG. Classification of stroke subtypes. Cerebrovasc Dis. 2009;27(5):493-501.

6. Hart RG, Diener HC, Coutts SB, Easton JD, Granger CB, O'Donnell MJ, et al. Embolic strokes of undetermined source: the case for a new clinical construct. Lancet Neurology. 2014;13(4):429-38.

7. Althunayyan A, Petersen SE, Lloyd G, Bhattacharyya S. Mitral valve prolapse. Expert Rev Cardiovasc Ther. 2019;17(1):43-51.

8. Andreasen C, Gislason GH, Kober L, Abdulla J, Martinsson A, Smith JG, et al. Incidence of Ischemic Stroke in Individuals With and Without Aortic Valve Stenosis: A Danish Retrospective Cohort Study. Stroke. 2020;51(5):1364-71.

9. Debette S, Grond-Ginsbach C, Bodenant M, Kloss M, Engelter S, Metso T, et al. Differential features of carotid and vertebral artery dissections: the CADISP study. Neurology. 2011;77(12):1174-81.

10. Jennette JC, Falk RJ, Bacon PA, Basu N, Cid MC, Ferrario F, et al. 2012 revised International Chapel Hill Consensus Conference Nomenclature of Vasculitides. Arthritis and rheumatism. 2013;65(1):1-11.

11. Headache Classification Committee of the International Headache Society (IHS) The International Classification of Headache Disorders, 3rd edition. Cephalalgia. 2018;38(1):1-211.

**Online supplementary table 3:** Subgroup RMS&OM etiologies

| **RMS** | **OM** |
| --- | --- |
| Hypercoagulable neoplastic | 4/6 (67%) AF  3/6 (50%) Atherosclerotic disease  1/6 (17%) PFO  1/6 (17%) Low ejection fraction <30% |
| Hypercoagulable non neoplastic | 2/5 (40%) PFO  1/5 (20%) AF  1/5 (20%) Low ejection fraction <30%  1/5 (20%) Atherosclerotic disease |
| Endocarditis | 4/5 (80%) AF  1/5 (20%) Dissection |
| Periprocedural | 2/6 (33%) AF  1/6 (17%) Atherosclerotic disease  3/6 (50%) Low ejection fraction <30% |
| Vasculopathy | 1/3 (33%) AF  2/3 (67%) Low ejection fraction <30% |
| Vasculitis | 1/1 (100%) Atherosclerotic disease |
| Vasospasm | 1/1 (100%) Cardiac mechanical valve |

**Online supplementary table 4**: Univariate comparison of the mixed etiology (RMS&OM) and “pure” RMS subgroups. Significant results of the univariate comparison are highlighted with an asterisk “*”.

| **Variable** | **RMS&OM (n= 27)** | **Pure RMS (n= 195)** | **OR** | **OR - 95% CI** | **p-value** |
| --- | --- | --- | --- | --- | --- |
| Age* | 71.9 (15.8) | 63.1 (23.3) | 0.96 | 0.93 – 0.99 | 0.017* |
| Sex (female) | 10 / 27 (37.0%) | 81 / 195 (41.5%) | 1.21 | 0.53 – 2.77 | 0.656 |
| NIHSS | 7.0 (12.5) | 7.0 (12.0) | 1.00 | 0.99 – 1.02 | 0.620 |
| Rankin before stroke | 0.0 (1.0) | 0.0 (1.0) | 1.29 | 0.82 – 2.03 | 0.273 |
| Functional outcome at 3 months (median mRs) | 3.0 (4.2) | 2.0 (5.0) | 0.97 | 0.80 – 1.18 | 0.768 |
| Favorable outcome at 3 months (mRs 0-2) (N, %) | 11 / 24 (45.8%) | 102 / 191 (53.4%) | 1.35 | 0.58 – 3.17 | 0.485 |
| Mortality at 12 months (N, %) | 7 / 24 (29.2%) | 63 / 183 (34.4%) | 1.27 | 0.50 – 3.24 | 0.609 |
| Stroke recurrence at 12 months (N, %) | 6 / 23 (26.1%) | 41 / 155 (26.5%) | 1.02 | 0.38 – 2.76 | 0.970 |

**Online supplementary table 5**: Additional demographic, clinical, and risk factor data in the study population. An asterisk highlights statistically significant factors in the univariate.

| **Variables** | **Overall population included** | **Rare stroke mechanisms**  **(N = 222)** | **All other mechanisms**  **(N = 3923)** | **OR (95% CI)** | **p-value** |
| --- | --- | --- | --- | --- | --- |
| **Clinical variables** |  |  |  |  |  |
| Previous ischemic stroke or TIA | 1113 / 4151 (26.8%) | 77 / 222 (34.7%) | 1036 / 3929 (26.4%) | 1.50* (1.12 - 1.99) | 0.016 |
| **Treatment before stroke onset** | | | | | |
| Any antiplatelet treatment | 1566 / 4147 (37.8%) | 79 / 221 (35.7%) | 1487 / 3926 (37.9%) | 0.91 (0.69 - 1.21) | 0.524 |
| Any anticoagulation | 448 / 4146 (10.8%) | 19 / 220 (8.6%) | 429 / 3926 (10.9%) | 0.77 (0.46 - 1.21) | 0.272 |
| Antihypertensive | 2455 / 4141 (59.3%) | 104 / 220 (47.3%) | 2351 / 3921 (60.0%) | 0.60* (0.46 - 0.79) | <0.01 |
| Lipid-lowering medication | 1185 / 4151 (28.5%) | 70 / 221 (31.7%) | 1115 / 3930 (28.4%) | 1.17 (0.87 - 1.56) | 0.295 |
| Any antidiabetic | 510 / 4146 (12.3%) | 21 / 221 (9.5%) | 489 / 3925 (12.5%) | 0.74 (0.45 - 1.14) | 0.178 |
| **Stroke risk factors** | | | | | |
| Prosthetic valves | 145 / 4149 (3.5%) | 15 / 222 (6.8%) | 130 / 3927 (3.3%) | 2.12* (1.17 - 3.56) | 0.015 |
| Low ejection fraction (<35%) | 229 / 4136 (5.5%) | 7 / 220 (3.2%) | 222 / 3916 (5.7%) | 0.55 (0.23 - 1.09) | 0.091 |
| Documented coronary artery disease | 693 / 4140 (16.7%) | 31 / 222 (14.0%) | 662 / 3918 (16.9%) | 0.80 (0.53 - 1.16) | 0.244 |
| Symptomatic peripheral artery disease | 262 / 4135 (6.3%) | 16 / 220 (7.3%) | 246 / 3915 (6.3%) | 1.17 (0.67 - 1.92) | 0.566 |
| Obesity | 1994 / 4136 (48.2%) | 102 / 222 (45.9%) | 1892 / 3914 (48.3%) | 0.91 (0.69 - 1.19) | 0.428 |
| Alcohol abuse | 420 / 4128 (10.2%) | 25 / 221 (11.3%) | 395 / 3907 (10.1%) | 1.13 (0.72 - 1.71) | 0.571 |
| Active oncological condition | 219 / 4138 (5.3%) | 44 / 221 (19.9%) | 175 / 3917 (4.5%) | 5.32* (3.66 - 7.59) | <0.01 |
| **Comorbidities** | | | | | |
| Migraine | 176 / 4114 (4.3%) | 8 / 222 (3.6%) | 168 / 3892 (4.3%) | 0.83 (0.37 - 1.60) | 0.601 |
| Myocardial infarction | 440 / 4145 (10.6%) | 19 / 221 (8.6%) | 421 / 3924 (10.7%) | 0.78 (0.47 - 1.23) | 0.303 |
| Any cardiac arrhythmia | 1590 / 4144 (38.4%) | 44 / 221 (19.9%) | 1546 / 3923 (39.4%) | 0.38* (0.27 - 0.53) | <0.01 |
| Any valvular disease (native or prosthetic valves) | 1030 / 4144 (24.9%) | 58 / 220 (26.4%) | 972 / 3924 (24.8%) | 1.09 (0.79 - 1.47) | 0.597 |
| Clinical congestive heart failure | 765 / 4143 (18.5%) | 34 / 221 (15.4%) | 731 / 3922 (18.6%) | 0.79 (0.54 - 1.14) | 0.215 |
| Recent weight loss | 30 / 4143 (0.7%) | 5 / 220 (2.3%) | 25 / 3923 (0.6%) | 3.63* (1.21 - 8.82) | 0.024 |
| Renal failure | 576 / 4144 (13.9%) | 29 / 221 (13.1%) | 547 / 3923 (13.9%) | 0.93 (0.61 - 1.37) | 0.729 |
| Fluid and electrolytic disturbance | 211 / 4143 (5.1%) | 14 / 221 (6.3%) | 197 / 3922 (5.0%) | 1.28 (0.70 - 2.16) | 0.404 |
| Acquired immune deficiency syndrome | 16 / 4144 (0.4%) | 4 / 220 (1.8%) | 12 / 3924 (0.3%) | 6.04* (1.68 - 17.49) | <0.01 |
| Blood-loss anemia | 66 / 4143 (1.6%) | 6 / 221 (2.7%) | 60 / 3922 (1.5%) | 1.80 (0.69 - 3.88) | 0.210 |
| Deficiency anemia | 264 / 4144 (6.4%) | 34 / 221 (15.4%) | 230 / 3923 (5.9%) | 2.92* (1.95 - 4.25) | <0.01 |
| Coagulopathy | 141 / 4145 (3.4%) | 28 / 221 (12.7%) | 113 / 3924 (2.9%) | 4.89* (3.10 - 7.48) | <0.01 |
| Lymphoma | 21 / 4145 (0.5%) | 2 / 221 (0.9%) | 19 / 3924 (0.5%) | 1.88 (0.30 - 6.52) | 0.437 |
| Metastatic cancer | 95 / 4142 (2.3%) | 27 / 221 (12.2%) | 68 / 3921 (1.7%) | 7.89* (4.86 - 12.46) | <0.01 |
| Solid cancer | 485 / 4142 (11.7%) | 43 / 220 (19.5%) | 442 / 3922 (11.3%) | 1.91* (1.34 - 2.68) | <0.01 |
| Drug abuse | 79 / 4141 (1.9%) | 14 / 221 (6.3%) | 65 / 3920 (1.7%) | 4.01* (2.13 - 7.05) | <0.01 |
| Psychosis | 358 / 4117 (8.7%) | 26 / 219 (11.9%) | 332 / 3898 (8.5%) | 1.45 (0.93 - 2.17) | 0.102 |
| Depression | 193 / 4112 (4.7%) | 22 / 219 (10.0%) | 171 / 3893 (4.4%) | 2.43* (1.49 - 3.80) | <0.01 |
| Dementia | 449 / 4135 (10.9%) | 8 / 221 (3.6%) | 441 / 3914 (11.3%) | 0.30* (0.13 - 0.56) | <0.01 |

**Online supplementary table 6**: Acute physiological, radiological and laboratory features in the study population. Statistically significant factors in the univariate analysis are highlighted by an asterisk.

| **Variables** | **Overall population included** | **Rare stroke mechanisms**  **(N = 222)** | **All other mechanisms**  **(N = 3923)** | **OR (95% CI)** | **p value** |
| --- | --- | --- | --- | --- | --- |
| **Acute physiological values** | | | | | |
| Rankin pre-stroke >2 | 429 / 4142 (10.4%) | 17 / 220 (7.7%) | 412 / 3922 (10.5%) | 0.71 (0.42 - 1.15) | 0.171 |
| Acute temperature (°Celsius) | 36.3 (0.7) | 36.4 (0.9) | 36.3 (0.7) | 1.46* (1.19 - 1.79) | <0.01 |
| Acute systolic blood pressure (per 10 mmHg) | 15.2 (3.5) | 14.1 (3.6) | 15.2 (3.4) | 0.85* (0.80 - 0.90) | <0.01 |
| Acute diastolic blood pressure (per 10 mmHg) | 8.4 (2.2) | 7.7 (2.0) | 8.4 (2.3) | 0.78* (0.72 - 0.85) | <0.01 |
| Acute heart ratio (beats per min.) | 78.0 (22.0) | 80.0 (21.5) | 78.0 (22.0) | 1.00 (1.00 - 1.01) | 0.241 |
| **Radiological variables** | | | | | |
| Early ischemic changes on CT-scan | 1973 / 3402 (58.0%) | 108 / 173 (62.4%) | 1865 / 3229 (57.8%) | 1.22 (0.89 - 1.67) | 0.223 |
| Symptomatic hemorrhagic transformation on subacute imaging | 95 / 4038 (2.4%) | 14 / 216 (6.5%) | 81 / 3822 (2.1%) | 3.25* (1.74 - 5.66) | <0.01 |
| Any leukoaraiosis | 1213 / 3719 (32.6%) | 35 / 187 (18.7%) | 1178 / 3532 (33.4%) | 0.46* (0.31 - 0.66) | <0.01 |
| Chronic stroke lesions | 1255 / 3719 (33.7%) | 60 / 187 (32.1%) | 1195 / 3532 (33.8%) | 0.92 (0.67 - 1.26) | 0.621 |
| Any arterial pathology (ischemic territory) | 1973 / 3402 (58.0%) | 108 / 173 (62.4%) | 1865 / 3229 (57.8%) | 1.22 (0.89 - 1.67) | 0.223 |
| Intracranial arterial pathology (ischemic territory) | 1792 / 3405 (52.6%) | 100 / 172 (58.1%) | 1692 / 3233 (52.3%) | 1.26 (0.93 - 1.73) | 0.136 |
| Extracranial arterial pathology (ischemic territory) | 777 / 3392 (22.9%) | 27 / 173 (15.6%) | 750 / 3219 (23.3%) | 0.61* (0.39 - 0.91) | 0.015 |
| **Acute laboratory variables** | | | | | |
| White blood cell count (Giga/liter) | 8.1 (3.7) | 8.6 (4.7) | 8.0 (3.6) | 1.03* (1.01 - 1.06) | <0.01 |
| Hemoglobin concentration (grams/liter) | 139.0 (23.0) | 129.0 (29.8) | 139.0 (22.0) | 0.97* (0.96 - 0.98) | <0.01 |
| Platelet count (per 10^8^/liter) | 22.4 (8.3) | 20.0 (9.1) | 22.3 (8.4) | 0.98* (0.96 - 1.00) | 0.025 |
| Glycemia (millimoles/liter) | 6.5 (2.2) | 6.5 (1.9) | 6.5 (2.2) | 0.96 (0.90 - 1.01) | 0.152 |
| Creatinine concentration (milligram/liter) | 87.0 (32.0) | 79.5 (31.0) | 87.0 (31.0) | 1.00* (0.99 - 1.00) | 0.039 |
| Total cholesterol concentration (millimoles/liter) | 5.1 (1.7) | 4.9 (1.9) | 5.1 (1.6) | 0.82* (0.71 - 0.95) | <0.01 |

mmHg= millimeters of Mercury
